# Predicting self-harm and suicide ideation during the COVID-19 pandemic in Indonesia: a nationwide survey report

**DOI:** 10.1186/s12888-022-03944-w

**Published:** 2022-04-29

**Authors:** Andrian Liem, Benny Prawira, Selvi Magdalena, Monica Jenifer Siandita, Joevarian Hudiyana

**Affiliations:** 1 Jeffrey Cheah School of Medicine and Health Sciences, Monash University Malaysia, Subang Jaya, Selangor Malaysia; 2 Into the Light Indonesia, Jakarta, Indonesia; 3 Faculty of Psychology, Universitas Indonesia, Depok, Jawa Barat Indonesia

**Keywords:** Suicide ideation, Health inequity, Mental health surveillance, Loneliness, Marginalized group, Minority, LGBTIQ

## Abstract

**Background:**

It is estimated that 77.0% of suicide cases occurred in low-and-middle-income countries (LMICs), which would increase because of the COVID-19 pandemic and socioeconomic inequity. However, there is lack of reports on this topic from LMICs, especially during the pandemic. Therefore, this nationwide study aimed to explore self-harm and suicide ideation and its predictive variables during the pandemic in Indonesia as a MIC with the highest COVID-19 fatality rate in Asia.

**Methods:**

Non-random sampling online survey was conducted nationwide between 25 May and 16 June 2021. The collected data were demographic variables (i.e. age group), loneliness from social isolation using The UCLA Loneliness Scale Six Items (ULS-6), and self-harm and suicide ideation using item 9 of The Patient Health Questionnaire-9 (PHQ-9). Predictive model was analyzed using hierarchical logistic regression.

**Results:**

A total of 5211 participants from all 34 provinces in Indonesia completed the survey. Among 39.3% of them reported self-harm and suicide ideation during the pandemic, which significantly correlated with loneliness. The predictive variables associated with the likelihood of self-harm and suicide ideation were age, residence, job, religion, sex-gender, sexual orientation, HIV status, disability status, and loneliness. The predictive model showed a significant goodness-of-fit to the observed data (*x*^2^ [ (15)] = 1803.46, *p* < .001), *R*_*N*_^2^ = .40.

**Conclusion:**

Four out of 10 Indonesians experienced self-harm and suicide ideation during the COVID-19 pandemic, particularly people within the age range of 18–24, living in the Java Island, unemployed/student/retired and freelancer, women, members of minority and marginalized communities, and experience of loneliness during the pandemic.

**Supplementary Information:**

The online version contains supplementary material available at 10.1186/s12888-022-03944-w.

## Highlights

• Four in 10 Indonesians experienced self-harm and suicide ideation during the pandemic.

• Loneliness from social isolation correlated with self-harm and suicide ideation.

• Socioeconomic inequity may contribute to self-harm and suicide ideation.

**Supplementary Information:**

The online version contains supplementary material available at 10.1186/s12888-022-03944-w.

## Background

Approximately 800,000 people worldwide died by suicide in 2019, 77.0% of whom occurred in low-and-middle-income countries (LMICs) [1], which was anticipated to increase due to the Coronavirus disease 2019 (COVID-19) pandemic. Life problems during this pandemic have been escalating and leading to mental disorders and self-harm, which is linked to suicide plan and suicide attempt [2–4]. A systemic review and meta-analysis on the prevalence of the symptoms of depression, anxiety, and stress during the COVID-19 pandemic in another LMIC like Bangladesh found the pooled prevalence of these three conditions were 47, 47, and 45% respectively [5]. Despite high suicide prevalence in LMICs, studies on this topic are clustered in high-income countries (HICs) and upper MICs. Therefore, the results might be less relevant to LMICs like Indonesia due to incomparable factors such as socioeconomic, resources, and health infrastructure between the two groups [6–8]. In addition, Indonesia was downgraded back as a lower-middle income country again after a year classified as a upper-middle income country in 2020 because the economy was affected by the COVID-19 pandemic [9].

Previous nationwide study on anxiety conducted early in the pandemic in Indonesia was found about 20% of participants reported clinical anxiety symptoms, mostly among younger people, unemployed, and women [10]. This finding is comparable to a nationwide survey in Bangladesh where 33% of the population were screened for depression, particularly among younger people [4]. A community study in Indonesia among men who have sex with men (MSM) and transgender individuals in July–September 2020 found that nearly 70% of participants reported worse psychological distress during the COVID-19 pandemic than pre-pandemic [11]. One of the reasons was discrimination faced by LGBTIQ people, which has existed long before the pandemic [12, 13]. In other studies, vulnerable and marginalized communities (e.g. people with HIV positive and people with disability) reported more severe mental problems than general population due to multiple barriers they faced during the pandemic [14–16]. Additionally, the burden was also experienced by the caregivers as found in a qualitative study among family members of schizophrenia patients who care them on a daily basis and felt more difficult to provide care during the pandemic, which also affected caregivers’ quality of life [17]. Despite the importance of the findings, these nationwide quantitative studies did not investigate suicidal behavior.

There are studies on suicide behavior in certain age groups and specific geographical locations in Indonesia in pre-pandemic [18–20]. However, there are no studies on self-harm and suicide ideation for wider population and more diverse groups, especially during the pandemic. Therefore, the current study aimed to explore self-harm and suicide ideation during the COVID-19 pandemic in Indonesia that had the highest COVID-19 fatality rate in Asia [21] and previous systematic analysis of suicide mortality burden found that the rates of suicide mortality in Indonesia was very high and influenced the average suicide rate the Southeast Asia region even before the pandemic [22]. Moreover, self-harm was identified as a predictor of suicides among young people in LMICs [23]. In addition, loneliness was consistently found to increase during the pandemic, which was associated with mental health problems and suicide attempt in diverse socioeconomic backgrounds globally [24–29]. This study will also give attention to demographic variables, minority status (i.e. sexual orientation), and loneliness from social isolation, as possible predictors of self-harm and suicide ideation during the COVID-19 pandemic in Indonesia. It is expected that findings from this study will contribute to suicide intervention plan and program, which more than half of the WHO member states are lacking, including Indonesia [1].

## Methods

### Participants and procedure

The participant inclusion criteria were Indonesian, aged 18 or older, and living in Indonesia. The survey was set up using Typeform online survey software. The software recorded 15,184 view times (only at the cover page) and 10,430 attempts to fill it in. At the end, a total of 5211 participants completed the survey data collection and incomplete responses were automatically deleted by the software. The response and completion rates could not be calculated as view times and attempts to fill in the online survey were not representing the real number of people. For example, one person could visit the cover page multiple times then filled in the survey but discontinued or paused it. When the person tried to fill it in again, previous data was deleted and the software counted it as a new attempt. The participants were predominantly aged 18–24 (45.9%), had a diploma or higher educational background (54.4%), were Muslims (70.9%), and identified as women (67.3%). Details of the participants were provided in the Results and total participants in each province is provided in Supplement 1. The data were collected online from 25 May to 16 June 2021 using non-randomized sampling method. The link to complete the online survey was posted on social media accounts of Into The Light (ITL) Indonesia and Change.org Indonesia^1^. The survey link was also sent through the mailing lists of ITL Indonesia and Change.org. To improve the survey inclusivity, personalized messages were sent to social media accounts of the marginalized communities (LGBTIQ people, people with disability, and people with HIV positive). This study was approved by the Research Ethics Committee of Research and Community Service Center (LPPM)-Universitas Katolik Indonesia Atma Jaya (0519A/III/LPPM-PM.10.05/05/2021).

### Measures

All items were presented in Bahasa Indonesia, including the demographic information: age groups, current province of residence, ethnicity, educational background, job status/type, income category, religion/belief, and sex-gender. There were also three questions that asked participants about their minority and marginalized status, namely sexual orientation, HIV status, and disability status that covered both mental and physical disabilities. The demographic variables were grouped in analysis by considering the sociocultural situation in Indonesia. For example, 34 provinces were categorized into two groups, namely “Outside Java Island” and “Within Java Island” because more than half of total population resided in six provinces (Banten, West Java, DKI Jakarta, Central Java, DI Yogyakarta, and East Java) in Java Island [30].

Loneliness from social isolation was measured using The UCLA Loneliness Scale Six Items (ULS-6)-Indonesian version [31], which has an *α* of .81 in this study. The ULS-6 is a cross-cultural measure of loneliness, and it primarily assesses the perception of objective social isolation. Four intensity options were provided for each item from Never (1) to Often (4). The total score ranged from 6 to 24, where higher score indicated more intense loneliness in the last 4 weeks. Participants who answered ‘Never’ on all six items were categorized as “never felt loneliness”, while participants who gave any other responses were grouped in “felt loneliness” for the descriptive analysis. The loneliness intensity was used for the correlational test with self-harm and suicide ideation intensity in the logistic regression analysis.

Self-harm and suicide ideation was measured using item number 9 of The Patient Health Questionnaire-9 (PHQ-9)-Indonesian version [32], which is “Over the last two weeks, how often have you been bothered by thoughts that you would be better off dead or of hurting yourself in some way?” with response options ranging from Not at all (0) to Nearly every day [3]. Previous study found that higher score means higher risk for suicide in mental health screening for the community sample [33]. For the descriptive and logistic regression analyses, participants who answered ‘Not at all’ were categorized in “no ideation”, while participants who answered any other responses were categorized in “with ideation”. The PHQ-9 has been validated among Indonesians, and the item 9 was classified into cognitive/affective factor of the two-factor model structure [34].

### Statistical analysis

Data analyses were conducted in Statistical Package for the Social Sciences (SPSS) version 26. Descriptive analysis was performed, followed by a Pearson’s test to analyze the correlation between loneliness intensity and self-harm and suicide ideation. A hierarchical logistic regression analysis was conducted to determine variables that predict self-harm and suicide ideation. Three blocks were built with the demographic variables in Block 1, minority conditions (sexual orientation, HIV status, and disability status) in Block 2, and loneliness intensity in Block 3. In order to explore the best model, all variables in three blocks were entered using the “Backward-Stepwise” method and the first category of each categorical variable was set as the indicator reference. Adjusted odds ratios with the associated 95% confidence intervals were reported. The significance value (*p*) was set at .05 (two-sided).

## Results

Table 1 showed that almost all participants (98.0%) reported feeling lonely during the pandemic. The proportion was relatively lower among participants who were aged 35 or older (95.1%), worked as civil servants/police/military (95.2%), and had monthly income of Rp 9,000,000-11,999,999 (≈USD 632–842) (95.5%). The intensity of loneliness was relatively less intense among groups of participants aged 35 or older and worked as civil servants/police/military than other groups. In contrast, participants with disability and participants who were HIV positive reported higher intensity.

**Table 1:** Demographic and statistics information (*N* = 5211)

Demographic variables	%	Loneliness experience (% yes)	Loneliness intensity (***M, SD***)	Self-harm & suicide ideation (% yes)	Self-harm & suicide ideation intensity (***M, SD***)
All participants	100	98.0	14.0 (4.6)	39.3	0.5 (0.8)
Age group					
18–24	45.9	98.9	15.2 (4.4)	50.4	0.7 (0.9)
25–34	34.0	98.5	13.8 (4.4)	39.1	0.5 (0.8)
≥ 35	20.1	95.1	11.3 (3.9)	14.2	0.2 (0.5)
Province					
Outside Java Island	24.2	97.5	13.7 (4.5)	36.1	0.5 (0.8)
Within Java Island	75.8	98.1	14.0 (4.6)	40.3	0.6 (0.8)
Ethnicity					
Non-Javanese	55.6	98.1	13.8 (4.5)	38.5	0.5 (0.8)
Javanese	44.4	97.9	14.1 (4.6)	40.3	0.6 (0.8)
Educational background					
High school and lower	45.6	98.6	14.8 (4.5)	45.2	0.7 (0.9)
Diploma and higher	54.4	97.5	13.2 (4.4)	34.3	0.5 (0.7)
Job status/type					
Unemployed/student/retired	41.1	98.6	15.3 (4.4)	50.4	0.7 (0.9)
Freelancer	13.9	98.1	13.8 (4.7)	39.4	0.5 (0.8)
Private employer/blue collar/driver/domestic worker	27.6	98.0	13.2 (4.4)	33.4	0.4 (0.7)
Civil servants/police/military	5.6	95.2	11.9 (4.2)	17.9	0.2 (0.6)
Entrepreneur	7.7	97.3	12.3 (4.1)	25.1	0.4 (0.7)
Others	4.1	96.2	12.7 (4.6)	22.6	0.3 (0.7)
Average monthly income group					
Other/have no income	9.8	98.8	15.2 (4.4)	50.9	0.7 (0.9)
< Rp 3,000,000 (≈ USD 210)	48.6	98.5	14.7 (4.5)	44.9	0.7 (0.9)
Rp 3,000,000-5,999,999 (≈ USD 210–421)	21.2	97.6	13.3 (4.4)	34.2	0.4 (0.7)
Rp 6,000,000-8,999,999 (≈ USD 422–631)	8.8	97.0	12.6 (4.4)	28.6	0.4 (0.7)
Rp 9,000,000-11,999,999 (≈ USD 632–842)	4.3	95.5	12.0 (4.0)	26.0	0.3 (0.6)
≥ Rp 12,000,000 (≈ USD 843)	7.3	97.4	12.0 (4.1)	21.5	0.3 (0.6)
Religion/belief					
Non-Islam	29.1	97.9	13.6 (4.4)	39.6	0.5 (0.8)
Islam	70.9	98.0	14.1 (4.6)	39.2	0.5 (0.8)
Sex-gender					
Male	31.9	96.8	12.9 (4.5)	28.6	0.4 (0.7)
Female	67.3	98.5	14.4 (4.5)	44.1	0.6 (0.8)
Intersex/transgender/non-binary	0.8	97.7	14.7 (4.7)	55.8	1.0 (1.0)
Sexual orientation					
Non-heterosexual	10.4	99.1	15.3 (4.5)	56.8	0.8 (0.9)
Heterosexual	89.6	97.9	13.8 (4.5)	37.2	0.5 (0.8)
HIV status					
Negative/do not know	99.2	98.0	13.9 (4.6)	39.1	0.5 (0.8)
Positive	0.8	100	15.5 (4.3)	62.8	0.9 (1.0)
Disability status					
Without disability	77.7	97.5	13.3 (4.4)	32.0	0.4 (0.7)
With disability	22.3	99.7	16.4 (4.3)	64.5	1.0 (1.0)

*M* Mean, *SD* Standard Deviation

More than one-third of the total participants (39.3%) reported self-harm and suicide ideation during the pandemic, which was relatively higher in some groups, including participants with disability (64.5%), participants with HIV positive (62.8%), and non-heterosexual participants (56.8%). Conversely, the percentage was relatively lower in some other groups, such as participants who were aged 35 or older (14.2%), worked as civil servants/police/military (17.9%), and had monthly income of USD 843 or higher (21.5%). In general, participants reported having self-harm and suicide ideation intensity nearly several days in the last 2 weeks (score 1 on the scale), which was relatively more intense among participants who identified themselves as intersex, transgender, or non-binary and participants with disability compared to other groups. Correlational test showed loneliness intensity was significantly and positively correlated with self-harm and suicide ideation intensity (*r* = .51, *p* < .001).

The final predictive model for self-harm and suicide ideation (Model 3) showed a significant goodness-of-fit to the observed data (*x*^2^ (15) = 1803.46, *p* < .001). Nagelkerke’s pseudo *R*^2^ indicated the significant variables in the third model explained approximately 40% of the variability. Variables and groups that were significantly associated with higher odds of experiencing self-harm and suicide ideation were age (18–24 years old), current residence (provinces in Java Island), job status/type (unemployed/student/retired and freelancer), religion (non-Islam), sex-gender (female), sexual orientation (non-heterosexual), disability status (with disability), and loneliness intensity (see Table 2).

**Table 2:** Hierarchical logistic regression models predicting self-harm and suicide ideation (*N* = 5211)

Variables	Model 1			Model 2			Model 3		
	Adj *OR*	95% *CI*	*p*	Adj *OR*	95% *CI*	*p*	Adj *OR*	95% *CI*	*p*
Age group									
18–24	1 (ref)			1 (ref)			1 (ref)		
25–34	0.81	0.70–0.94	***	0.80	0.68–0.93	**	0.91	0.77–1.09	.30
≥ 35	0.24	0.19–0.29	***	0.26	0.21–0.33	***	0.39	0.31–0.50	***
Province									
Outside Java Island	1 (ref)			1 (ref)			1 (ref)		
In Java Island	1.33	1.16–1.53	***	1.29	1.12–1.50	**	1.24	1.06–1.46	**.01**
Job status/type									
Unemployed/student/retired	1 (ref)			1 (ref)			1 (ref)		
Freelancer	0.92	0.76–1.11	.38	0.88	0.72–1.08	.22	0.99	0.80–1.24	.98
Private employer/blue collar/driver/domestic worker	0.68	0.58–0.81	***	0.70	0.59–.83	***	0.82	0.68–0.99	**.04**
Civil servants/police/military	0.39	0.28–0.54	***	0.41	0.29–0.59	***	0.53	0.36–.077	**
Entrepreneur	0.62	0.48–0.81	***	0.64	0.48–0.84	**	0.85	0.63–1.14	.27
Others	0.42	0.30–0.60	***	0.42	0.29–0.61	***	0.47	0.31–.071	***
Religion/belief									
Non-Islam	1 (ref)			1 (ref)			1 (ref)		
Islam	0.79	0.69–0.91	**	0.84	0.73–0.96	**.01**	0.75	0.65–0.88	***
Sex-gender									
Male	1 (ref)			1 (ref)			1 (ref)		
Female	1.55	1.35–1.77	***	1.70	1.47–1.97	***	1.55	1.32–1.82	***
Intersex/transgender/non-binary	2.49	1.30–4.74	**	1.43	0.73–2.79	.29	1.53	0.73–3.17	.26
Sexual orientation									
Non-heterosexual				1 (ref)			1 (ref)		
Heterosexual				0.49	0.39–0.60	***	0.58	0.46–0.73	***
HIV status									
Negative/do not know				1 (ref)			1 (ref)		
Positive				2.31	1.16–4.60	**.02**	1.90	0.92–3.95	.08
Disability status									
Without disability				1 (ref)			1 (ref)		
With disability				3.25	2.81–3.75	***	2.18	1.86–2.56	***
Loneliness intensity							1.27	1.25–1.29	***
Constant	0.76		**	0.99		.93	0.03		***
Overall correct predicted percentage	63.5			68.1			75.2		
*R*_*N*_^2^	.14			.22			.40		
* Δ R*_*N*_^*2*^	.14			.08			.18		
* Omnibus test / Chi-square (df)*	566.99 (10)***			921.23 (14)***			1803.46 (15)***		

*Adj OR* Adjusted Odd Ratio, *CI* Confidence Interval, *Ref* Reference category. ** = *p* < .01. *** = *p* < .001. RN2 = Nagelkerke’s pseudo R-squared. Δ = Delta. df Degree of freedom

## Discussion

This exploratory study aimed to investigate self-harm and suicide ideation and its predictive variables during the COVID-19 pandemic in Indonesia. Findings from this study complemented previous studies on suicidal behavior in specific age groups and limited geographical locations in Indonesia before the pandemic [18–20]. It was found that nearly 40% of participants in this study reported self-harm and suicide ideation. The ideation intensity in this study was relatively low (*M* = 0.5, *SD* = 0.8), but it was higher than previous study that used the same item among female Indonesian migrant workers in Macau (*M* = 0.1, *SD* = 0.3) [34]. This difference illustrated how pandemic affected psychological conditions because the data in the Macau study was not collected during a pandemic. Furthermore, the current prevalence was relatively higher compared to a longitudinal study that also used the same instrument among young people in Hong Kong, whereby 21% of the participants reported self-harm and suicide ideation in June 2020 [3]; and also higher among Bangladeshi people who were only 5% of them answering “yes” on a question “Do you think about committing suicide, and are these thoughts persistent and related to COVID-19 issues?” in April 2020 [4].

The final predictive model showed that the younger age group (18–24 years old) had significantly 2.56 times higher chance of experiencing self-harm and suicide ideation than the older group (35 years or older). Our findings supported another representative population study among Indonesian but it was on anxiety, which found older age participants have lower risk of developing the disorder during the pandemic [10]. We also found 50.4% of the young participants aged 18 to 24 reported having self-harm and suicide ideation, which was higher than suicidal ideation among Indonesian adolescent students and young adults before the pandemic [19, 20, 35]. The higher odds among young participants might be associated with challenges in transitioning to adulthood and adapting to online learning during the pandemic, which differed from their prior expectations [2, 11]. Furthermore, previous studies found young generation accessed COVID-19 related information primarily from social media, which could lead to infodemic and triggered COVID-19 infection anxiety [10, 36].

Participants within the six provinces in Java Island, where more than 56% of total Indonesians resided [30], were predicted to have a significant 1.24 times greater chance of experiencing self-harm and suicide ideation than citizens in other areas. This might be because provinces in Java had been severely impacted by COVID-19 with the highest infection rate, more so than other provinces during the two different pandemic waves [37]. Also, the infrastructure and access inequity within Java Island and disparity with other islands could be another reason. For example, 66.7% of the Class A hospitals with the best facilities and resources are located in Java Island, but they are much more accessible by people from high-income background, whereas people with low-and-middle income have limited resources (e.g. cannot take day off work and difficult transportation access) [21, 38]. Moreover, participants in this study might be originally from outside Java and came to the island for studying or working, as evidenced by the domestic migration rate to six provinces in Java Island that reached 50.3% of total internal migration in Indonesia [39]. Consequently, these domestic migrants might live alone and often do not have adequate social support, particularly young adults. This could become a risk factor as previous studies found family support could buffer the psychological problems during the crisis like the pandemic [2, 3, 40]. The current study also aligned with a previous study on suicidal behavior among Indonesian people before the pandemic, whereby people living in urban area had 3.00 times higher suicide risk due to economic stress than people living in non-urban area [18].

Unemployed and freelancer groups showed greater chance of having self-harm and suicide ideation, which was up to 1.89 times higher when compared with members of civil servant, police, or military group. This finding mirrored previous nationwide studies that also found unemployed participants were 4.78 times more likely to experience anxiety than health workers in Indonesia [10]. The pressure as an economic-provider for these unemployed participants might be the reason for this psychological condition, as found in other countries with high unemployment rates [40]. The self-harm and suicide ideation percentage was also found higher among participants with lower income, which is consistent with a systematic review on the association between economic inequity and poorer mental health, particularly in LMICs [7].

People with a minority-religion background significantly had 1.33 times greater odds of experiencing self-harm and suicide ideation than Muslim participants, which has the largest population in Indonesia (86.9%) [41]. Movement restriction during the pandemic have made individuals unable to visit their worship places and meet their fellow believers. This could be a contributing factor to psychological problems given religious and spirituality aspects could not be detached from the sociocultural lives of Indonesians [42, 43]. Moreover, individuals belonging to religious minority affiliations do not have as much support and feel more isolated from the mainstream culture [44]. On the other hand, Muslim participants also could not attend mosques, however, their religious and spiritual connection could still be maintained because it is common for Indonesian mosques to use loud-speakers, which the sound (*Adzan*) is considered as an extension of a mosque in practicing the Islamic teachings (*dakwah*) and for religious purposes like a call to prayer and preaching (*khutbah*) [45]. Moreover, the loudspeaker is also used for non-religious functions in daily lives like obituary news and community service (*gotong royong*) events.

Compared to men, women had 1.55 times higher likelihood for experiencing self-harm and suicide ideation. This trend was also found in an anxiety predictive model in Indonesia during the pandemic, where women had significantly 1.61 times higher odds of developing clinically significant anxiety than men [10]. The reason might be because women faced more life challenges than men during the pandemic, primarily related to preconception and fertility, including unplanned pregnancy, perinatal stress, postpartum stress and depression, and miscarriage [46]. Moreover, compared to men, women in the patriarchal society are expected to be more active in parenting and caregiving tasks, with additional risks of experiencing domestic violence [47]. Consequently, within these more challenging situations, women were more likely developed mental disorders such as anxiety and depression that were consistently found to be associated with suicidal behavior [3, 18].

An alternative explanation is men in the current study might be less open in expressing their feelings and mental problems because they worry about being perceived as weak and less masculine if they admit their struggles [27, 40]. Previous study also found that objective living alone condition was more significant in triggering self-harm and suicidal ideation than loneliness among men [28]. This condition was reflected by men’s loneliness experience percentage and intensity that were relatively lower than women in the current study.

Three predictive models were developed where the *R*_*N*_^2^ increased from .14 in Model 1 to .40 when participant’s minority conditions (sexual orientation, HIV status, and disability status) and loneliness intensity were included in Model 3, which became the novelty of this study by discovering the contribution of minority conditions and loneliness intensity to individual’s self-harm and suicide ideation. It was found in the final model that non-heterosexual participants (10.4% of total participants) were significantly more likely to experience self-harm and suicide ideation, 1.72 times than heterosexual people. On the other hand, intersex/transgender/non-binary people also had higher odds in all three predictive models (2.49, 1.43, and 1.53 respectively), but the odds were statistically non-significant in the final model. This might be because this group was under-represented in the sample (0.8% of total participants).

These findings were consistent with a study among Indonesian transgender individuals and MSM that found 69.1% of the participants experienced moderate to high level of psychological distress during the pandemic that increased the risk of suicide behavior [11]. Moreover, members of intersex/transgender/non-binary community lived like invisible citizens as their existence was not recognized by the government. Many of them were forced to have no citizen identity card, which means they were unable to access public services and aids. During the COVID-19 pandemic, many of them died without being surrounded by any relatives, and had to be buried without a tombstone because their identity was unknown [48].

People with positive HIV status who are also part of minority and marginalized communities had 1.90 times greater odds in the final model for experiencing self-harm and suicide ideation. However, this finding was statistically non-significant that might be due to the low representation in the overall participants (0.8%). Despite the non-significant ratio, the current finding reinforced previous studies that highlighted the very limited access to mental health services for LGBTIQ and people living with HIV worldwide, particularly in LMICs, which has worsened during the pandemic [13, 14].

People with disability were significantly predicted to have 2.18 times higher chance of experiencing self-harm and suicide ideation than people without disability. This could be because the pandemic had adversely impacted social interactions, ability to study, and opportunity to earn an income for people with disability, more severely than people without disability. For example, mask-wearing policy as part of COVID-19 health protocols meant people with deaf and hard of hearing were unable to read lip movements and communicate well with others [16]. Moreover, inadequate support for people with disability may also contribute to their deteriorating mental health. For instance, sign language interpreter was not provided during the COVID-19 press conferences, so deaf people would find it extremely difficult to understand the updated situation and regulations regarding the pandemic [49]. In addition, a community study in the US found that COVID 19-related stress was significantly associated with maladaptive coping strategies, including denial, substance use, and self-blame among people with disabilities and chronic conditions, which resulted in declining well-being [15].

Intensity of loneliness from social isolation among participants in this study was higher (*M* = 14.0, *SD* = 4.6) compared with the ULS-6 validation study in Indonesia, Germany, and the US, where Indonesian participants reported the lowest score (*M* = 12.4, *SD* = 3.83) [31]. In the final predictive model, loneliness contributed to 18.0% of variability. The model predicted that loneliness from social isolation would increase the chance of people experiencing self-harm and suicide ideation by 1.27 times. These findings were in line with a longitudinal study during the pandemic (May–July 2020) in Brazil, where loneliness significantly increased the odds of suicidal ideation by 2.12 times fold [29].

The current study also found that prevalence of loneliness due to social isolation among younger participants was higher than the older group. These young participants might be dominated by students who studied away from their hometown and lived alone, which would amplify loneliness experience. Furthermore, chronic loneliness was consistently found to be associated with psychological disorders, particularly depression, among children, adolescents, and young adults worldwide [25]. However, loneliness was statistically non-significant associated with how many people were around the individual, or the duration of conversation they had [27]. In support of this, a longitudinal study in the UK found that chronic loneliness in adolescents during the COVID-19 pandemic, not how often they talked with friends, was significantly correlated with deteriorating mental health and the relationship was bidirectional [24]. Therefore, more social interventions that address loneliness from social disconnectedness need to be targeted especially to the young age group.

### Study strengths and limitations

The important strengths in this study were the large nationwide participants sampled from all provinces in Indonesia with diverse backgrounds and the use of validated instruments. However, there was an over-representation of participants who lived in Java Island that might have different experiences from people in other provinces outside Java Island. Also, despite the data being collected from members of minority and marginalized communities, their numbers were relatively small and might be insufficient for statistical analyses. The question of sex and gender was also mixed due to the public negative sensitivity towards gender nonconformity. Next, this online cross-sectional study does not allow for causal interpretations. The results were also limited to the data collection period and may have the risk of self-selection bias. Lastly, despite the PHQ-9 has been validated among Indonesians this instrument was originally designed to screen for depression; and used a single item (Item 9) to screen self-harm and suicide ideation in non-primary care situations might be less accurate compared with other instruments that are specially designed for this purpose. Therefore, future study should reach participants from outside of Java Island and members of minority and marginalized communities and use a measure with higher sensitivity and specificity for screening suicide ideation in a general setting. Findings from this quantitative study should also be complemented by qualitative investigation, particularly the experiences of minority and marginalized communities that might not be suitable to be analyzed statistically.

## Conclusion

Four in ten people in Indonesia may experience self-harm and suicide ideation during the COVID-19 pandemic. Variables that significantly contributed to predicting self-harm ideation were age, province, job status, religion, sex, sexual orientation, disability status, and intensity of loneliness from social isolation. The likelihood of experiencing self-harm and suicide ideation were higher among people aged 18–24, lived in Java Island, unemployed/student/retired and freelancer, women, members of minority and marginalized communities (non-Islam, non-heterosexual, and with disability), and those experiencing loneliness from social isolation during the pandemic. Mental health treatment and suicide prevention programs during and post-pandemic should prioritize these high-risk groups and be tailored by involving the group members in the planning processes.

## Supplementary Information

**Additional file 1.**

## Data Availability

The Indonesian version of survey methods and results can be accessed from the Into The Light Indonesia website^2^. Derived data supporting the findings of this study are available from the corresponding author on request.

## Footnotes

1 ITL Indonesia is a youth-based community with the focus as a center of advocacy, research, and education related to suicide prevention and mental health issues in Indonesia. Change.org Indonesia is the Indonesian office of Change.org, an American petition website that offers the public the ability to promote the petitions they care about to potential signers.
