# Supplementary material for: Predicting self-harm and suicide ideation during the COVID-19 pandemic in Indonesia: a nationwide survey report
Source: BMC Psychiatry. 2022 Apr 29;22:304. doi: 10.1186/s12888-022-03944-w (PMC9051849; doi:10.1186/s12888-022-03944-w)
Supplement: Supplementary file 1 — Additional file 1. [file 12888_2022_3944_MOESM1_ESM.docx]

# Full-title

Predicting self-harm and suicide ideation during the COVID-19 pandemic in Indonesia: A nationwide survey report

**Short-title**

Self-harm & suicide ideation during the pandemic

**Type**

Original research article

**Authors and affiliations**

1. Andrian Liem*

Jeffrey Cheah School of Medicine and Health Sciences, Monash University Malaysia, Selangor, Malaysia; andrian.liem@monash.edu; https://orcid.org/0000-0002-1746-7235.

2. Benny Prawira*

Into the Light Indonesia, DKI Jakarta, Indonesia; prawirabenny89@gmail.com; https://orcid.org/0000-0003-4417-9674.

3. Selvi Magdalena

Into the Light Indonesia, DKI Jakarta, Indonesia; selvi.eru@gmail.com.

4. Monica Jenifer Siandita

Into the Light Indonesia, DKI Jakarta, Indonesia; jensiandita@gmail.com.

5. Joevarian Hudiyana

Faculty of Psychology, Universitas Indonesia, Jawa Barat, Indonesia; joevarian91@ui.ac.id; https://orcid.org/0000-0001-5507-0573.

**corresponding authors*

# SUPPLEMENT

Supplement 1. Total participants in each province

| **No** | **Province** | ***n*** | **%** |
| --- | --- | --- | --- |
| 1 | Jawa Barat | 1,172 | 22.49 |
| 2 | DKI Jakarta | 1,012 | 19.42 |
| 3 | Jawa Timur | 585 | 11.23 |
| 4 | Jawa Tengah | 509 | 9.77 |
| 5 | Banten | 431 | 8.27 |
| 6 | DI Yogyakarta | 243 | 4.66 |
| 7 | Sumatera Utara | 154 | 2.96 |
| 8 | Bali | 148 | 2.84 |
| 9 | Sulawesi Selatan | 81 | 1.55 |
| 10 | Kalimantan Timur | 77 | 1.48 |
| 11 | Lampung | 68 | 1.30 |
| 12 | Sumatera Barat | 67 | 1.29 |
| 13 | Riau | 60 | 1.15 |
| 14 | Sumatera Selatan | 59 | 1.13 |
| 15 | Kalimantan Barat | 58 | 1.11 |
| 16 | Nusa Tenggara Timur | 56 | 1.07 |
| 17 | Kepulauan Riau | 55 | 1.06 |
| 18 | Kalimantan Selatan | 53 | 1.02 |
| 19 | DI Aceh | 46 | 0.88 |
| 20 | Sulawesi Utara | 43 | 0.83 |
| 21 | Nusa Tenggara Barat | 41 | 0.79 |
| 22 | Jambi | 33 | 0.63 |
| 23 | Sulawesi Tengah | 27 | 0.52 |
| 24 | Kalimantan Tengah | 26 | 0.50 |
| 25 | Bengkulu | 16 | 0.31 |
| 26 | Kepulauan Bangka Belitung | 16 | 0.31 |
| 27 | Sulawesi Tenggara | 15 | 0.29 |
| 28 | Gorontalo | 13 | 0.25 |
| 29 | Papua | 12 | 0.23 |
| 30 | Kalimantan Utara | 11 | 0.21 |
| 31 | Maluku | 10 | 0.19 |
| 32 | Papua Barat | 8 | 0.15 |
| 33 | Maluku Utara | 4 | 0.08 |
| 34 | Sulawesi Barat | 2 | 0.04 |
|  | Total | 5,211 | 100.00 |

*Note*. Provinces number 1-6 are in Java Island.
